# Reevaluating antibiotic prophylaxis: insights from a network meta-analysis on dry socket and surgical site infections

**DOI:** 10.1038/s41432-024-01067-7

**Published:** 2024-11-14

**Authors:** Tayebe Rojhanian, Ahmad Sofi-Mahmudi, Amin Vahdati

**Affiliations:** 1 Department of Community Oral Health, Faculty of Dentistry, Hormozgan University of Medical Science, Bandar Abbas, Iran; 2 Department of Health Research Methods, Evidence and Impact, McMaster University, Hamilton, ON Canada; 3 Department of National Pain Centre, Department of Anesthesia, McMaster University, Hamilton, ON Canada; 4 Centre for Biostatistics, School of Health Science, Faculty of Biology, Medicine and Health, University of Manchester, Manchester, UK

**Keywords:** Health care, Antibiotics in dentistry

## Abstract

**Data sources:**

Three databases (MEDLINE, Cochrane Library, and Scopus) were searched in December 2021 for 16 Randomised Clinical Trials (RCTs).

**Study selection:**

Three reviewers reviewed the articles on oral antibiotic prophylaxis (ABP) for the prevention of surgical site infection (SSI) and dry socket (DS) after lower third molar (L3M) extraction using the PICO framework. From 1999 to 2021, RCTs involving healthy patients undergoing L3M extraction with ABP, placebo, or no therapy were included. Adverse effects (AEs) associated with antibiotic usage, along with the main outcomes (DS and SSI), were also documented.

**Data extraction and synthesis:**

Three independent investigators selected articles based on pre-established inclusion criteria, with any disagreements resolved by consensus or additional researchers. PRISMA guidelines were followed, involving initial title and abstract screening, followed by full-text evaluation. Exclusion reasons were documented, and the most recent report was included when multiple reports on the same patients were found, with no language restrictions applied. Two investigators evaluated studies quality and quality of evidence respectively using the Cochrane Collaboration tool and GRADEpro GDT. They independently extracted data, focusing on the type of extraction and the number of extracted L3M. They also detailed the use of antibiotics, including dosage, dosage regimen, timing, and duration. Among 16 articles, 15 used a parallel arm design, while one used a crossover design. The antibiotics studied included Amoxicillin+Clavulanic acid (7 articles), Amoxicillin (6), Metronidazole (2), Azithromycin (1), and Clindamycin (2), all compared with no treatment or placebo. A pairwise meta-analysis was used to combine studies with equivalent treatment (direct estimation), and a network meta-analysis compared outcome variables across different treatments (indirect comparison).

**Results:**

Two included articles had a low risk of bias and the level of evidence was low according to GRADE. Pooled results supported the use of antibiotics to reduce DS and SSI following L3M extraction with a number needed to treat 25 and 18, respectively.

**Conclusions:**

Despite the fact that ABP reduces the risk of DS and SSI, it is recommended to consider systemic conditions and individual patient risk factors before prescribing antibiotics, due to global health threat.

## A Commentary on

**Camps-Font O, Sábado-Bundó H, Toledano-Serrabona J, Valmaseda-de-la-Rosa N, Figueiredo R, Valmaseda-Castellón E**

Antibiotic prophylaxis in the prevention of dry socket and surgical site infection after lower third molar extraction: a network meta-analysis. *Int J Oral Maxillofac Surg* 2024; **53:** 57–67.

**GRADE Rating:** 

## Commentary

Lower third molar (L3M) extraction is a common dental procedure under anesthesia^1^. However, it often comes with risks like dry socket (DS) and surgical site infection (SSI), which can cause discomfort and, in rare cases, severe infections that may be fatal. SSI incidence after third molar removal is about 30%, while DS incidence ranges from 0.5% to 30%^2,3^.

Antibiotic prophylaxis (ABP) has been proposed as a way to prevent DS and SSI^4^. While antibiotics can reduce the incidence of these complications, their use must be evidence-based. Inappropriate or unnecessary prescribing of antibiotics not only wastes healthcare resources but also contributes to the critical issue of antimicrobial resistance (AMR)^5,6^. It is essential to explore non-antibiotic strategies that could be effective in preventing these complications. Enhanced tooth extraction techniques, such as lessening tissue trauma and reducing extraction time -factors that are heavily influenced by the operator’s experience-can significantly reduce the risk of infection and dry socket^7^. Using antiseptic mouthwashes before and after procedure has also been suggested as helping to control bacterial load in the oral cavity^8–10^. By ensuring patients understand and adhere to post-operative care instructions, such as avoiding smoking and following appropriate oral hygiene practices, the likelihood of complications can be further minimized^9^. These strategies not only reduce the need for antibiotics but also empower patients to take an active role in their recovery, potentially leading to better outcomes and a reduction in the incidence of post-surgical complications.

This study analysed data from 16 randomized clinical trials involving 2158 patients. It assessed the effectiveness of antibiotics like Amoxicillin, Amoxicillin with Clavulanic acid, Metronidazole, Azithromycin, and Clindamycin compared to a no-treatment or placebo group. The results showed that ABP significantly reduces the risk of DS and SSI, with a number needed to treat (NNT) of 25 for DS and 18 for SSI. This means that while antibiotics are effective, many patients might receive them without direct benefit—only one in 25 or one in 18 patients treated with antibiotics will actually avoid DS or SSI, respectively. This highlights the need for targeted antibiotic use, reserving prophylaxis for patients at higher risk of complications, rather than applying it broadly to all patients undergoing L3M extraction. Such an approach can reduce unnecessary antibiotic prescriptions and help mitigate the risk of AMR.

The study appropriately focuses on DS and SSI as primary outcomes, given their clinical relevance^11^. However, the definitions and diagnostic criteria for DS and SSI vary across studies, leading to inconsistencies. Furthermore, this study failed to sufficiently consider the operator’s clinical proficiency in completing lower tooth extraction, a crucial aspect that greatly impacts the probability of developing dry socket following the extraction.

Most of the selected trials (13 out of 16) focused on Amoxicillin, either alone or combined with Clavulanic acid, reflecting the common use of this antibiotic in dental practice. This broad focus ensures the findings are relevant to typical clinical settings. However, the trials varied in the timing and combination of antibiotic administration (preoperative, postoperative, or both). This inconsistency could affect the validity of the SUCRA rankings, as combining different protocols might introduce variability that is not fully accounted for.

A significant limitation of the study is its inability to perform a network meta-analysis for adverse effects (AEs) due to high heterogeneity among the trials and insufficient data, with only seven out of sixteen studies evaluating AEs. This lack of comprehensive reporting introduces a notable reporting bias, likely leading to an underestimation of the true incidence of complications such as nausea, vomiting, diarrhea, and other mild AEs. The discussion on AEs is also limited and does not provide detailed insights into their frequency and severity, nor does it fully explore the implications in the specific context of dental antibiotic use.

While the study acknowledges the general safety of Amoxicillin and its broad spectrum of activity, it would benefit from a more focused and detailed analysis of AEs directly related to the use of antibiotics in preventing DS and SSI after L3M extraction. Future research should aim for more rigorous and standardized reporting of AEs to enhance the understanding of the risk-benefit profile of ABP in this clinical setting. Drafting guidelines for prescribing after extracting L3M, considering various aspects such as effectiveness, necessity, safety, and social impact, can prevent unnecessary antibiotic prescriptions and mitigate their detrimental effect on public health. It is imperative that dental practitioners consider the long-term implications for their prescribing habits, improve their understanding and awareness of AMR in dentistry, and resist the temptation to prescribe antibiotics under patient pressure^12,13^. By adhering to evidence-based practices, clinicians can play a critical role in curbing the spread of AMR.

The overall certainty of the evidence, as evaluated using the GRADEpro tool, was rated very low.

Practice points

- Antibiotic prophylaxis effectively reduce DS and SSI risk in healthy patients after L3M extraction, but the number needed to treat is high.
- Preoperative Clindamycin was most effective for preventing DS, while postoperative Amoxicillin was most effective for preventing SSI.
